# Distribution in Rat Blood and Brain of TDMQ20, a Copper Chelator Designed as a Drug-Candidate for Alzheimer’s Disease

**DOI:** 10.3390/pharmaceutics14122691

**Published:** 2022-12-01

**Authors:** Lan Huang, Yaoxun Zeng, Yongliang Li, Yingshan Zhu, Yan He, Yan Liu, Anne Robert, Bernard Meunier

**Affiliations:** 1 School of Chemical Engineering and Light Industry, Guangdong University of Technology, Guangzhou 510006, China; 2 School of Biomedical and Pharmaceutical Sciences, Guangdong University of Technology, Guangzhou 510006, China; 3 Laboratoire de Chimie de Coordination du CNRS (LCC-CNRS), Inserm ERL 1289, 205 Route de Narbonne, 31077 Toulouse, France

**Keywords:** Alzheimer’s disease, blood, brain, copper chelator, oxidative stress, pharmacokinetics

## Abstract

(1) Background: TDMQ20 is a specific regulator of copper homeostasis in the brain, able to inhibit cognitive impairment in the early stages of Alzheimer’s disease (AD) in mouse models of AD. To promote the further development of this drug-candidate, preliminary data on the pharmacokinetics of TDMQ20 in a mammal model have been collected. Since TDMQ20 should be administered orally, its absorption by the gastrointestinal tract was evaluated by comparison of blood concentrations after administration by oral and IV routes, and its ability to reach its target (the brain) was confirmed by comparison between blood and brain concentrations after oral administration. (2) Methods: plasmatic and brain concentrations of the drug after oral or intravenous treatment of rats at pharmacologically relevant doses were determined as a function of time. (3) Results: oral absorption of TDMQ20 was rapid and bioavailability was high (66% and 86% for males and females, respectively). The drug accumulated in the brain for several hours (brain–plasma ratio 3 h after oral administration = 2.6), and was then efficiently cleared. (4) Conclusions: these data confirm that TDMQ20 efficiently crosses the brain–blood barrier and is a relevant drug-candidate to treat AD.

## 1. Introduction

Alzheimer’s disease (AD) is one of the major public health issues in the world. With the increase in life expectancy, this age-related disease is creating considerable pressure on patients, families, caregivers and public health budgets. Unfortunately, despite tremendous efforts by universities and pharmaceutical companies to research it, very few new treatments have been approved since memantine in 2003 [1,2]. Only five drugs, four acetylcholine esterase (AChE) inhibitors (tacrine, donepezil, rivastigmine, galantamine) and a weak antagonist of the N-methyl-D-aspartate (NMDA) receptor (memantine), have been approved until recently. These drugs are not curative, offering only short-term symptomatic relief, a high level of side effects and questionable efficiency/cost ratios [3]. For these reasons, many national health insurance plans have decided to stop the reimbursement of these treatments. Moreover, the level of failure in clinical trials of new therapies, small molecules or biopharmaceuticals, is close to 100%. In recent years, only GV-917 (a sodium oligomannate) has been approved in China [4,5], and aducanumab (a monoclonal antibody targeting amyloid proteins) by the US FDA [6]. However, this latter biopharmaceutical drug has been rejected by the European agency [7].

Therefore, due to the complexity of the etiology of AD, there is an urgent need to explore other potential targets to enlarge the drug spectrum with different mechanisms, and to design new disease-modifying therapies to slow or stop the neurodegenerative process. The destruction of neurons in brains affected by AD has been attributed to an oxidative stress mainly caused by rupture of the homeostasis of redox-active metal ions, such as copper and iron [8,9]. As of 25 January 2022, among the 143 disease-modifying therapies involved in 172 clinical trials for AD (Phases I–III), only 3 of them (2%) targeted oxidative stress, but none of them were designed to restore redox metal homeostasis [10].

**Figure 1:**
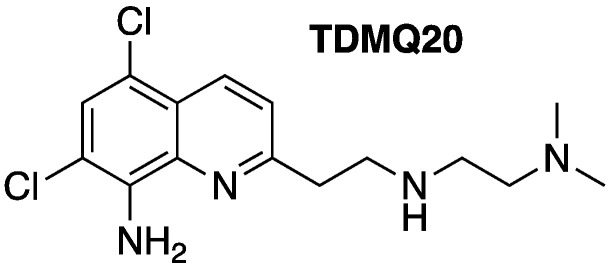
Structure of TDMQ20 copper chelator.

When not bound to copper-enzymes or copper-carrier proteins, free copper ions can be trapped to amyloids and, in the presence of endogenous reductants, can generate—via the redox cycle of Cu(II)/Cu(I)—the hydroxyl radicals, HO•, that are responsible for oxidative damage leading to neuron death [11]. Copper complexes of β-amyloid short oligomers, able to penetrate neurons, might trigger this oxidative stress within different neuronal sub-compartments [12]. Such a catalytic process causes an overconsumption of endogenous antioxidants, and thus their depletion in neurons. Moreover, the sequestration of copper in amyloid plaques may generate a copper deficit in other compartments of the brain and may reduce Cu,Zn-SOD activity [13]. The restoration of copper homeostasis by using specific chelators has therefore been considered a key pharmacological target [14,15]. Recently, we designed new N4-tetradentate ligands, named TDMQs, specifically for copper chelation, which are innocent with respect to copper enzymes or other metalloproteins [16,17,18]. These TDMQ ligands are able to extract copper from Cu-Aβ and inhibit the formation of ROS (reactive oxygen species), even in the presence of an excess of Zn(II) ions [19]. Within this series of specific copper chelators, TDMQ20 (see Figure 1 for structure) has been selected as drug-candidate and evaluated on three different murine models of Alzheimer’s disease, two non-transgenic ones (icv-CuAβ and hippo-CuAβ) and the usual transgenic one (5xFAD) [20,21]. Data obtained with these three murine models indicated that the oral administration of TDMQ20 to mice at 10 mg/kg was able to inhibit memory deficit, and reduced the formation of malondialdehyde in the brains of treated AD-mice [20]. The mechanism of action of TDMQ20 should be considered bi-modal, acting as (i) an inhibitor of ROS production due to its capacity to extract copper from copper amyloid (evidenced by the lower concentration of malondialdehyde in the brains of treated AD-mice [20]), and (ii) a provider of copper to copper-deprived cholinergic neurons [21]. The lack of acute or chronic toxicity of TDMQ20 upon oral administration to WT C57BL/6J mice and 5xFAD transgenic AD mice indicates that TDMQ20 is safe, even in long-term treatment of animals weakened by the disease (one dose of 10 mg/kg every two days for 3 months, total dose = 450 mg/kg) [18].

In order to document the pharmacological data on this lead molecule, we investigated the pharmacokinetics of TDMQ20 in rats and evaluated the concentration of TDMQ20 within the brains of treated rats. Since the oral route is obviously preferable for the comfort of patients in human treatment (specially to old, demented, multi-medicated patients), absorption of TDMQ20 by the gastrointestinal tract was evaluated by comparison of blood concentrations after oral and IV administration, and the ability of TDMQ20 to reach the brain, its target organ, was evidenced by comparison between blood and brain concentrations after oral administration. The results of this study are reported here.

## 2. Materials and Methods

### 2.1. Chemicals and Materials

TDMQ20 was synthesized as previously reported [16,17]. An amount of 2-methyl-8-nitroquinoline (purity > 98%), used as an internal standard (IS), was purchased from BidePharm (Shanghai, China). HPLC-grade methanol was purchased from Thermo Fisher Scientific (Shanghai, China). HPLC-grade acetonitrile was purchased from TEDIA (Anhui, China). Aqueous NaCl (0.9 wt%) was purchased from G-clone (Beijing, China).

### 2.2. LC–MS/MS Method

A triple-quadrupole mass spectrometer (Thermo Fisher, Waltham, MA, USA) was connected to a Thermo Fisher Ultimate 3000 HPLC system (Thermo Fisher, USA). Data collection was controlled by Thermo Scientific Xcalibur software. Samples were separated on a Hypersil GOLD C18 column (50 × 2.1 mm, 1.9 μm) at 40 °C. The mobile phase was a gradient of (A) 0.1% formic acid aqueous solution and (B) methanol. The gradient elution was as follows: from 0 to 1 min, A/B = 90/10, then a linear gradient from A/B = 90/10 at 1 min to A/B = 10/90 at 3 min, followed by 3 min (from 3 to 6 min) at A/B = 10/90 (Table S1). Flow rate: 0.3 mL/min. Injected volume: 1 μL. All analytes were quantified after optimization of MRM mass spectrometry, with detection in positive ion mode, with selected parameters and considered fragmentations detailed in Tables S2 and S3, respectively. The mass spectrum of TDMQ20 (*m*/*z* = 327.3, MH^+^) and the structures of its fragments with *m*/*z* = 239.2 and 204.2 are depicted in Figure S1. Due to its having the highest abundance, the transition 327.3 → 239.2 was used for TDMQ20 quantification. For the internal standard, 2-methyl-8-nitroquinoline was used (IS, *m*/*z* = 189.1, MH^+^), with fragments at *m*/*z* = 143.17 and 128.17. The transition 189.1 → 143.2 was used for IS quantification.

### 2.3. Preparation of Calibration and Quality Control Samples

The stock solution of TDMQ20 was prepared at a concentration of 3.0 mg/mL in 0.9 wt% aqueous NaCl. The stock solution of 2-methyl-8-nitroquinoline (IS) was prepared at a concentration of 3.0 mg/mL in methanol. A series of working solutions was prepared by diluting the TDMQ20 stock solution in 0.9% NaCl. The IS stock solution was further diluted to 2000 ng/mL and 8000 ng/mL in methanol. All standard solutions were stored at 4 °C before use. The calibration standards of TDMQ20 were prepared by spiking TDMQ20 working standard solutions (10 µL) into blank plasma and brain homogenate. Calibration curves were prepared at TDMQ20 concentrations of 10, 25, 50, 100, 200, 400, 800, 1000 ng/mL. Quality control (QC) samples were similarly prepared at four different levels in plasma at concentrations of 10, 25, 500, 750 ng/mL (namely, lower limit of quantification (LLOQ), low quality control (LQC), middle quality control (MQC), and high-quality control (HQC), respectively). According to guidelines on bioanalytical method validation from the EMA, FDA and CFDA, the lowest calibration standard (LLOQ), three times the LLOQ (LQC), around 30–50% of the calibration curve range (MQC), and at least 75% of the upper calibration curve range (HQC) were analyzed against the calibration curve, and the obtained concentrations were compared with nominal values [22,23,24].

### 2.4. Animal Groups

Six-to-seven-week-old Sprague–Dawley rats (220–300 g for males, 200–240 g for females) were purchased from Guangdong Medical Laboratory Animal Center (GDMLAC, Guangzhou, China) and kept 6 per cage (with males and females separated), at 22 ± 2 °C with a relative humidity of 55% ± 10% and an approximately 12 h light/dark cycle. After a 7-day acclimation period, rats were fasted for 12 h prior to experiments, except for free access to water. They were then assigned to groups as indicated in Table 1.

To summarize, groups 1 and 2 consisted of control animals (untreated) whose blood and brains were spiked with TDMQ20 before analysis. Groups 3M and 3F consisted of animals (males and females, respectively) treated by the oral route for dosage of TDMQ20 in plasma. Each animal was subjected to blood collection 9 times, between 10 min and 12 h after drug treatment. Groups 10M and 10F consisted of animals (males and females, respectively) treated by the intravenous route for dosage of TDMQ20 in plasma. Each animal was submitted to blood collection 9 times, between 2 min and 5 h after treatment. Groups 4M/F, 5M/F, 6M/F, 7, 8 and 9 consisted of animals (males and females, respectively, for 4M–6M and 4F–6F, and males for 7–9) treated by the oral route for dosage of TDMQ20 in the brain. Animals from these groups were killed and the brains were collected after 3 h, 5 h, 7 h, 12 h, 24 h and 48 h after drug treatment for groups 4M/F, 5M/F, 6M/F, 7, 8 and 9, respectively. Details are provided below.

Each of the controls (groups 1 and 2) consisted of 6 control (untreated) male rats. The blood of animals from group 1 (300 μL) was collected from the retro-orbital venous plexus. The animals from group 2 were anesthetized by intraperitoneal injection of 20% urethane (1500 mg/kg) and killed by cardiac perfusion with 0.9 wt% aqueous NaCl, and the brains were immediately collected. The control blood (group 1) and control brain tissue (group 2) were spiked with known amounts of TDMQ20 to validate the method of quantification of TDMQ20 (see below). Group 3M consisted of 6 male rats intragastrically fed a single dose of TDMQ20 in 0.9 wt% aqueous NaCl at 25 mg/kg (ca. 2 mL of solution). From each rat, blood samples (300 µL) were then collected from the retro-orbital venous plexus at fixed times (ranging from 10 min to 12 h) after treatment, in order to evaluate the concentration of TDMQ20 in rat plasma with respect to time after oral administration of the drug. Group 3F consisted of 6 female rats that were treated and analyzed in the same way as group 3M. These rats, from 3M and 3F, were then killed by cervical dislocation after urethane induced anesthesia (ip, 1500 mg/kg) 24 h after TDMQ20 treatment. Groups 4M, 5M and 6M each consisted of 6 male rats that were intragastrically fed a single dose of TDMQ20 in 0.9 wt% aqueous NaCl at 25 mg/kg. Animals were killed by cardiac perfusion with 0.9 wt% aqueous NaCl after 3 h, 5 h or 7 h for groups 4M, 5M or 6M, respectively. The brains were immediately collected to evaluate the concentration of TDMQ20 in rat brain homogenates with respect to time after oral administration of the drug. Groups 4F, 5F and 6F each consisted of 6 females, and were treated and analyzed in the same way as groups 4M, 5M and 6M, respectively. Groups 7, 8 and 9 each consisted of 6 male rats that were orally treated with TDMQ20 (25 mg/kg) in the same way as groups 4M–6M, and whose brains were analyzed 12 h, 24 h and 48 h, respectively, after drug treatment. Groups 10M and 10F consisted of 6 males and 6 females, respectively, that received a single intravenous injection of TDMQ20 (2.5 mg/kg in 0.9 wt% aqueous NaCl). The drug was dosed in blood samples collected from the retro-orbital venous plexus between 2 min and 300 min after treatment, as with groups 3–6. The size of each group (6 animals) was fixed at the minimum necessary to obtain statistically significant data. Each rat was labelled using an ear tag. A single analysis was performed for each sample (blood or brain) collection. Excel software was used to calculate the mean values and SEM (standard error of the mean) of the concentration of TDMQ20 in plasma and in the brain, and to plot this concentration with respect to time after treatment. DAS 2.0 software (BioGuider Co., Shanghai, China) was used to calculate pharmacokinetic data (Table 2).

### 2.5. Ethics Approval

All the animal welfare and experimental procedures were approved by the “Welfare and ethics of laboratory animals” committee of the Guangdong University of Technology (GDUT) (approval no. GDUTXS2022083), and were performed by Guangzhou Huateng Bioscience Co., Ltd, Guangzhou, China (accreditation no. SYXK-2020-0237), in accordance with relevant guidelines and regulations for the care and use of laboratory animals, including ARRIVE and BJP guidelines. Sprague–Dawley rats are outbred multipurpose laboratory animals, bred especially for physiology and CNS studies, and preclinical assays. In addition, they are docile and easy to handle, a feature that minimizes unsuccessful tests. So, all animals used and all samples collected were included in data analyses. The staff in charge of the animal experiments received appropriate training, and all efforts were made to minimize animal pain and discomfort. The number of animals used was limited to the strict minimum necessary to achieve scientific validity of the results, in the framework of the 3Rs principle.

### 2.6. Preparation of Plasma Sample and Brain Extract for Calibration

A total of 100 µL of plasma from each rat in group 1 was mixed with IS working solution (2000 ng/mL, 10 µL). The mixture was vortexed at 1500 rpm for 1 min. Methanol (100 µL) and acetonitrile (200 µL) were added for protein precipitation. The mixture was vortexed and centrifuged at 10,000 rpm for 10 min at room temperature. The supernatant (1 µL) was injected for LC–MS/MS analysis.

For brain samples, 1 g brain tissue from a single rat from group 2 was homogenized with 0.9 wt% NaCl (5 mL). Brain homogenate (200 µL) was mixed with IS working solution (8000 ng/mL, 10 µL). The mixture was vortexed and then methanol (200 µL) and acetonitrile (400 µL) were added for protein precipitation. Then, the mixture was vortexed and centrifuged at 10,000 rpm for 10 min at room temperature. The supernatant (1 µL) was injected for LC–MS/MS analysis.

### 2.7. Method Validation

Selectivity. The selectivity was investigated by comparing blank plasma and brain homogenates from six individual SD rats with the spiked plasma and brain homogenates at the lower limit of quantification (LLOQ) level of the analyte. The analytical method allowed us to distinguish TDMQ20 and IS from endogenous components in the matrix or other components in the sample [22,23,24].

Linearity of the mass spectrometry response with respect to TDMQ20 or IS concentration. LLOQ. The calibration curve was constructed by plotting the peak area ratio of TDMQ20/IS (i.e., the area of the transition 327.3 → 239.2 of TDMQ20 divided by the area of the transition 189.1 → 143.2 of IS) (Y) against TDMQ20 concentration (X) using a 1/X weighting factor. The lower limit of qualification was defined as the lowest TDMQ20 concentration of the calibration curve (10 ng/mL). The TDMQ20 calibration curve is depicted in Figure S2.

Accuracy and precision. The accuracy and precision of measures were determined by determination of TDMQ20 concentration in five replicates of LLOQ, LQC, MQC and HQC on three days [22,23,24]. The relative error (RE%), indicating accuracy of the measure, is calculated as [(measured concentration—spiked concentration)/spiked concentration] × 100. RE should be within ±15% of the LQC, MQC and HQC. The coefficient of variation (CV), indicating precision of the measure, is calculated as standard deviation divided by mean. CV should be lower than 15% of the LQC, MQC and HQC. For LLOQ, RE should be within ±20%, and CV should be <20%.

Extraction recovery and matrix effect. Extraction recovery was determined by comparing the peak area of TDMQ20 spiked in blank plasma and blank brain homogenate against TDMQ20 spiked in post-extracted blank plasma and brain homogenate in LQC and HQC concentrations. The matrix effect was determined by comparing the peak area of TDMQ20 spiked in post-extracted blank plasma and blank brain homogenate against TDMQ20 spiked in solvent. The recovery and matrix effect should not exceed 15% [22,23,24].

Stability of TDMQ20 in biological samples. The stability of TDMQ20 in plasma and brain homogenate was investigated by analysis of LQC and HQC samples of TDMQ20-spiked samples from untreated animals, stored at room temperature for 4 h, at 4 °C for 24 h, at −20 °C for 14 days and after three freeze–thaw cycles. The measured concentrations should be within ±15% of the nominal concentrations.

### 2.8. Pharmacokinetics and Brain Distribution of TDMQ20

Concentration of TDMQ20 in plasma. Six male and six female SD rats (groups 3M and 3F, respectively) were treated with a single oral dose of TDMQ20 (25 mg/kg). Blood samples (300 µL each) were collected from the retro-orbital venous plexus in EDTA anticoagulant tubes at 10, 20, 30, 60, 120, 300, 480 and 720 min after treatment. They were centrifuged at 8000 rpm for 10 min at 4 ℃; the supernatant plasma samples were then withdrawn and stored at −20 °C until analysis. Six male and six female rats (groups 10M and 10F, respectively) were treated with a single intravenous dose of TDMQ20 (2.5 mg/kg). Blood samples were collected at 2, 5, 10, 30, 60, 90, 120, 180 and 300 min after treatment. Each sample was treated and analyzed in the same way as after oral treatment (groups 3M and 3F, see above).

Concentration of TDMQ20 in brain extracts. Six groups of 6 male rats each were used (groups 4M, 5M, 6M, 7–9). Animals were treated by oral administration of TDMQ20 at a single dose of 25 mg/kg. At 3 h, 5 h, 7 h, 12 h, 24 h and 48 h after administration for groups 4M, 5M, and 6M, 7, 8 and 9, respectively, the brains were collected after cardiac perfusion with 0.9 wt% aqueous NaCl. Then, the tissues were washed with 0.9% NaCl, blotted with filter paper, weighed and stored at −20 °C until use. Groups 4F, 5F and 6F, consisting of 6 female rats each, were treated in the same fashion as groups 4M, 5M and 6M, respectively.

Data treatment. The plots of the concentrations of TDMQ20 in plasma and brain samples with respect to time after treatment were obtained using Excel software. The standard error of the mean (SEM), which measures how much discrepancy is likely in a sample’s mean compared with the population mean, was also calculated using Excel software. The pharmacokinetics parameters of TDMQ20 were calculated using non-compartmental methods by DAS 2.0 Software (Drug and Statistics, Shanghai University of Traditional Chinese Medicine, Shanghai, China). Statistical significance (unpaired *t*-test, Mann–Witney) was determined using GraphPad Prism 7 software (GraphPad Software, San Diego, CA, USA; ** stands for *p*-value < 0.01, * stands for 0.01 < *p*-value < 0.05).

## 3. Results and Discussion

### 3.1. Validation of the TDMQ20 Quantification Method

Selectivity of detected peaks for TDMQ20 and IS. In the LC–MS/MS conditions, the retention times of TDMQ20 and IS were 3.71 min and 3.85 min, respectively. In these conditions, no interfering peak was detected in typical chromatograms of plasma or brain extracts. Typical MRM chromatograms of blank plasma, blank plasma spiked with TDQM20 at LLOQ level or IS, and plasma samples collected at 1.0 h after oral administration of TDMQ20 are shown in Figure S3. Typical MRM chromatograms of blank brain homogenate, blank brain homogenate spiked with TDMQ20 at LLOQ levels or IS, and brain samples collected at 2 h after oral administration of TMDQ20 are shown in Figure S4.

Linearity of the LC–MS/MS response and LLOQ. By spiking known amounts of TDMQ20 or IS in the plasma and brain extract of untreated rats, calibration curves were constructed by comparing the peak area ratio of TDMQ20/IS (Y) (= ratio of peak area of the transition 327.3 → 239.2 (TDMQ20) divided by the peak area of the transition 189.1 → 143.2 (IS)) against TDMQ20 concentration (X) using the least squares linear regression method with 1/X weighting. The linear regression equations were Y = 0.00348153 X + 0.00298342 and Y = 0.0162424 X + 0.00171123 for plasma and brain homogenate, respectively. The calibration curves displayed good linearity (r > 0.99) over the range 10–1000 ng/mL (Figure S2). The LLOQ of TDMQ20 was the lowest measured concentration: 10 ng/mL. Accuracy and precision of the calibrations were within ±20% of RE and RSD.

Accuracy and precision of TDMQ20 dosage. The results of intra-day and inter-day accuracy and precision are shown in Table S4. The accuracy, expressed as RE, ranged from −5.9% to 10.2%, and the precision, expressed as coefficient of variation (CV), was within 14.5% (Table S4). The accuracy and precision were within the acceptable range for the bio-sample analysis, confirming that the method was reliable and reproducible.

Extraction recovery and matrix effect. The extraction recoveries for LQC and HQC samples were 91% and 95%, respectively (Table S5), indicating that the endogenous substances of the plasma and brain tissue do not affect the recovery and quantification of TDMQ20.

Stability of TDMQ20 in spiked samples. Quantification of TDMQ20 in plasma and brain homogenate took place at D0 and after storage or treatment in four different conditions: room temperature for 4 h, HPLC auto-sampler for 12 h, −20 °C for 14 d, and three freeze–thaw cycles. In all cases, the measured concentrations were in the acceptable range [22,23,24]. These results demonstrated that TDMQ20 can be considered stable in these biological media under these storage conditions (Table S6).

### 3.2. Quantification of TDMQ20 in Rat Plasma after Intravenous Administration

While the oral route is obviously preferable for human treatment (especially for old, demented, multi-medicated patients), animal studies using the IV route are necessary to investigate PK parameters, especially bioavailability. In this respect, twelve healthy adult Sprague–Dawley rats (male/female ratio = 1/1, animal groups 10M and 10F, respectively) were treated by the intravenous route with a single dose of TDMQ20 (2.5 mg/kg; about 0.5 mg per animal). After the treatment with TDMQ20, blood was collected from the retro-orbital venous plexus over a 5 h period. Quantification of TDMQ20 in plasma was then carried out from 2 min to 5 h by tandem mass spectrometry (MS/MS, MRM mode). Fragmentations of TDMQ20 molecular ions (*m*/*z* = 327.1, MH^+^) produced fragments at *m*/*z* = 239.1 and 204.1 (Figure S1). For the internal standard, 2-methyl-8-nitroquinoline was used (IS, *m*/*z* = 189.1, MH^+^), with fragments at *m*/*z* = 143.2 and 128.12.

**Figure 2:**
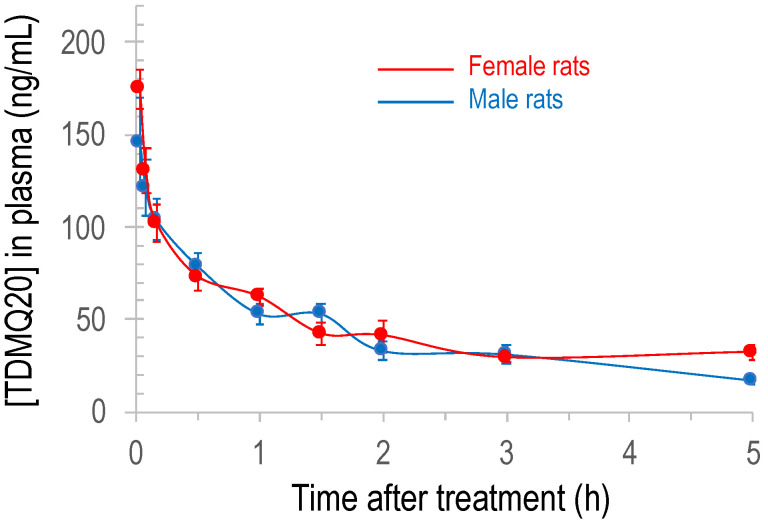
Plot of the concentration of TDMQ20 detected by LC–MS/MS in rat plasma as a function of time after intravenous administration of the drug (2.5 mg/kg). Mean values of 6 female and 6 male rats (red and blue traces, respectively), bars stand for SEM.

The plot of the concentration of TDMQ20 detected by LC–MS/MS in rat plasma with respect to time after IV administration of the drug is depicted in Figure 2 (individual rat data are provided in Table S7). The plots for male rats (blue trace) and female rats (red trace) are clearly superimposable, showing that there is no significant difference between males and females. The detected dose in plasma at 2 min was in the range 146–175 ng/mL, far higher than the validated limit of detection of 10 ng/mL. It quickly decreased, being −64–65% and −77–78% at 1 h and 2 h, respectively, compared to the concentration measured at 2 min. Thereafter, the decrease in TDMQ20 concentration was much slower, its concentration being −82–88% at 5 h, compared to the concentration measured at 2 min after administration (divided by 1.3–1.9 between 2 h and 5 h) (Table S7). So, the blood collection was stopped at 5 h after collecting eight blood samples per animal. On the basis of these experimental data (0–5 h), pharmacokinetic parameters were extracted using the Drug and Statistics (DAS) version 2.0 software; the results are summarized in Table 2.

### 3.3. Quantification of TDMQ20 in Rat Plasma after Oral Administration

Twelve healthy adult Sprague–Dawley rats (male/female ratio = 1/1, groups 3M and 3F, respectively) were treated by the oral route with a single dose of TDMQ20 (25 mg/kg, about 5 mg per animal). After treatment, blood was collected from the retro-orbital venous plexus over a 12 h period, and quantification of TDMQ20 in plasma was then carried out from 10 min to 12 h.

**Figure 3:**
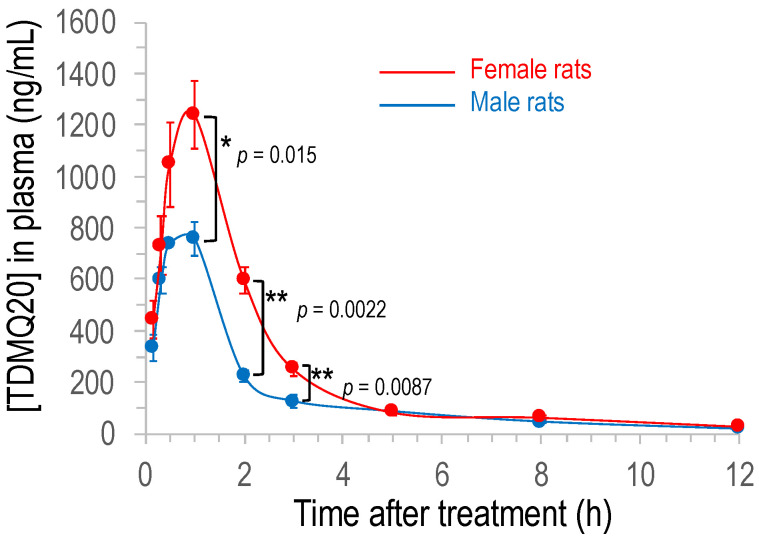
Plot of the concentration of TDMQ20 detected by LC–MS/MS in rat plasma as a function of time after oral administration of the drug (25 mg/kg). Mean values of 6 female and 6 male rats (red and blue traces, respectively), bars stand for SEM. The *p*-values were determined using unpaired *t*-test (Mann–Whitney); ** stands for *p*-value < 0.01, * stands for 0.01 < *p*-value < 0.05.

The plot of the concentration of TDMQ20 detected in rat plasma with respect to time after oral administration of the drug is depicted in Figure 3 (individual rat data are provided in Table S8). It is noteworthy that the detected concentration of TDMQ20 in plasma after oral administration was significantly higher in female than in male rats, between 1 h and 3 h (*p*-values 0.015, 0.0022 and 0.0087, respectively). The concentrations before 1 h and after 4–5 h were not statistically different in males and females (*p* = 0.24 or 0.94 at 30 min and 5 h, respectively).

In both cases, the concentration quickly increased after oral administration of the drug, to reach the maximum calculated concentration C_max_ of 830 μg/L and 1251 μg/L for males and females, respectively (corresponding to 2.5 μM and 3.8 μM, respectively) after 0.6–0.8 h (Table 2), indicating a quick oral absorption of TDMQ20 in rats. Elimination from plasma was also rapid with an elimination half-time t_1/2_ in the range 2.7–3.3 h. The AUC_0−∞_ was 1.84 mg/L·h for males and 3.05 mg/L·h for females, and the apparent oral clearance CL/F was 14 L/h/kg and 9 L/h/kg for males and females, respectively (Table 2). The bioavailability of TDMQ20 after oral treatment was high, 66% and 86% in males and females, respectively.

Sex differences in drug PK/PD have long been reported in animals and in humans (see, for example, [25,26]). In general, various physiological sex differences that are clearly known can significantly modulate the absorption (different enzymatic equipment of digestive tract, gastric fluid volume and pH, different gut transit times), distribution (different regional blood flow), metabolism (sex-related expression of CYP450) and elimination (sex-differences in all major renal function) of drugs. In the present case, since the PK of TDMQ20 after IV administration was similar in males and females (see above), the sex difference of C_max_ and bioavailability after oral administration of the drug might be due to sex differences in digestive absorption or first-pass metabolism.

### 3.4. Quantification of TDMQ20 in Rat Brain after Oral Administration

**Figure 4:**
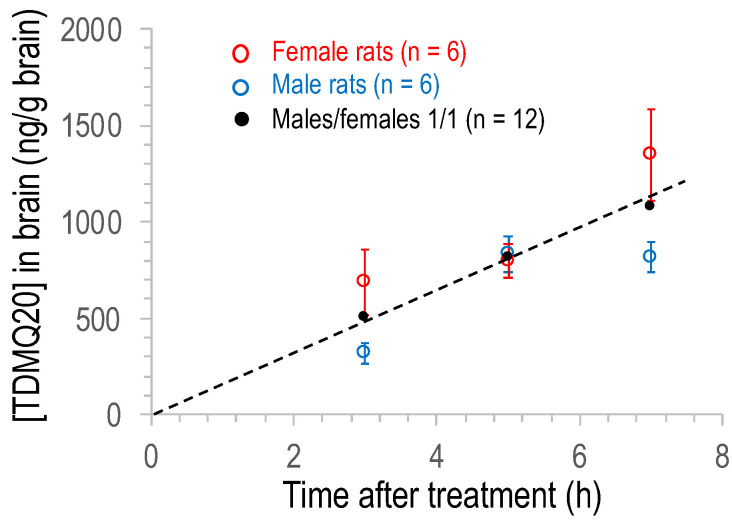
Plot of the concentration of TDMQ20 detected by LC–MS/MS in brain homogenates of male (blue) or female (red) or male/female, 1/1 (black) rats as a function of time after oral administration of the drug (25 mg/kg). Each dot is the mean value of 6 males, 6 females or 12 rats (male/female ratio = 1/1). Vertical bars stand for SEM values.

TDMQ20 was also quantified in brain homogenates between 3 h and 48 h after a single oral treatment at 25 mg/kg. The mean concentration of TDMQ20 detected in the brains of males and females in the range 3–7 h after oral administration of the drug (groups 4–6), is plotted in Figure 4 (individual rat data are reported in Table S9). The differences between males (blue marks) and females (red marks) were not statistically significant (*p*-values = 0.093, 0.937 and 0.132 at 3 h, 5 h and 7 h, respectively), in contrast to the concentration measured in plasma (Figure 3). More importantly, considering the mean value of TDMQ20 concentration for all 12 of the treated animals (male/female ratio = 1/1, black dots), TDMQ20 progressively accumulates in the brain over at least 5–7 h, long after it decreased from plasma (ca. 3 h). This might be due to an intermediate storage that was not investigated further (for instance, in the gallbladder). The mean concentration in the brain (male/female 1/1) was ca. 1083 ± 266 ng/g after 7 h, higher than the C_max_ detected in plasma (830 ng/mL). The brain concentration of TDMQ20 at 7 h (1083 ng/g) corresponds to 3.3 nanomoles/g of brain (MW of TDMQ20 base = 326 g/mol) or a total amount of TDMQ20 in the brain of ca. 2 μg. It is noteworthy that this concentration is on the same order of magnitude as Aβ_1-42_ concentration in the cortical frontal area of patients with AD (1.9 nmole/g [27]). This concentration is also higher than the high nanomolar range of Aβ_1-42_ concentration considered deleterious for neuronal signaling in vitro [28].

**Figure 5:**
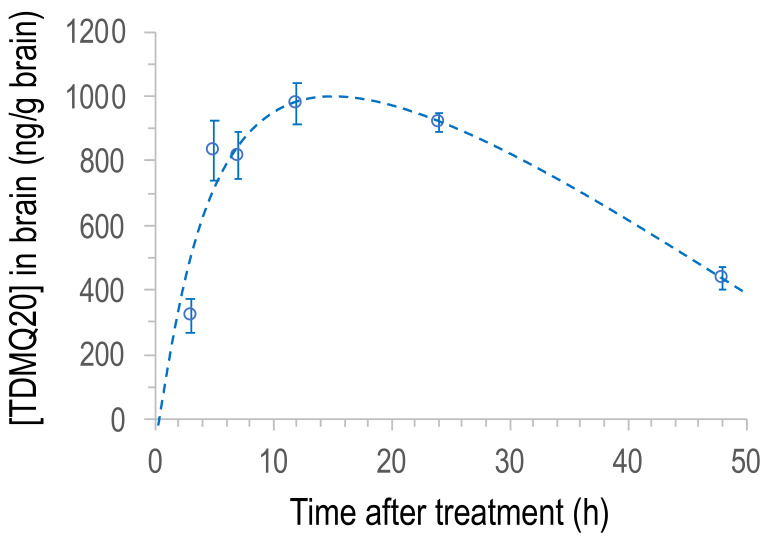
Plot of the concentration of TDMQ20 detected by LC–MS/MS in brain homogenates of male rats as a function of time after oral administration of the drug (25 mg/kg). Each dot is the mean value of 6 animals. Vertical bars stand for SEM values.

In order to evaluate the clearance of the drug from the brain, the concentration of TDMQ20 in the brains of male rats was also measured at 12, 24 and 48 h after a single oral dose of 25 mg/kg (groups 7, 8 and 9, respectively). Since the concentration of TDMQ20 in the brains of males and females was not statistically different at 3 h, 5 h and 7 h, continuation of the experiment on both sexes was neither scientifically nor ethically justified. So, we decided to continue the experiment at 12, 24 and 48 h only with males. The results obtained with male rats in the range 3–48 h are reported in Figure 5. The concentration of the drug in the brain increased up to ca. 1100 ng/g at 13–15 h after administration, and then decreased by roughly 56% between 15 h and 48 h ([TDMQ20] = 439 ng/g after 48 h). These results indicate that TDMQ20 accumulates in the brain for several hours, consistent with its expected catalytic activity of copper transport from amyloid to proteins. It was then eliminated indicating a slow but efficient clearance of the drug from the brain within a few days. In addition, this kinetic of brain elimination is consistent with the administration frequency (3 times per week) we have used to evaluate its ability to inhibit AD brain impairment [20,21].

The brain–plasma concentration ratio (also referred to as brain–blood ratio) is an important tool to evaluate the brain-targeting efficiency of potential neurotherapeutics, and drugs having a brain–plasma ratio value close to 1 are considered to freely cross the blood–brain barrier (BBB) [29,30]. The brain–plasma ratio of TDMQ20 concentration was evaluated to be ca. 2.6–2.8 at 3 h after drug administration (see footnote of Table S9). These data confirm the ability of TDMQ20 to cross the BBB and its efficient penetration within brain. This is consistent with its physicochemical properties that meet the criteria for a CNS-pharmaceutical drug [29,30] (MW = 326 g/mol (<450), clogP = 2.5 (<7), polar surface area [31] = 53.65 Å^2^ (60–70 Å^2^), hydrogen bond donors and hydrogen bond acceptors = 3 and 4, respectively (<3 and 7, respectively), 6 rotatable bonds (<8) and pKa values of the amine side chain in the range 7.5–10.5).

## 4. Conclusions

After oral administration of TDMQ20 to rats at 25 mg/kg, the drug was detected both in plasma and in the brain at micromolar concentration. Its half-life in plasma is relatively short (3 h) but its high bioavailability (66% and 86% for males and females, respectively) allowed for a good distribution of the drug in rats, indicating that TDMQ20 can be efficiently administered by the oral route. Moreover, TDMQ20 very efficiently accumulates in the brain, its target organ, during the first 15 h, until reaching a concentration (ca. 1100 ng/g in male brains) in the same range as the C_max_ value detected in plasma (830 ng/mL and 1251 ng/mL in males and females, respectively), followed by an appropriate clearance period. The TDMQ20 concentration in the brain is within the same order of magnitude as pathological amyloid concentrations. This fact supports the ability of this copper chelator to extract copper from amyloids within the brain. Taking into consideration the present pharmacokinetics data and the previously collected pharmacological data, TDMQ20 can be considered as a qualified drug-candidate for the treatment of Alzheimer’s disease.

## Figures and Tables

**Table 1:** Rat groups for dosage of TDMQ20 in plasma and brain after a single intragastric (25 mg/kg) or intravenous (2.5 mg/kg) administration of the drug.

Group Number	Number of Rats	Sex	Treatment			Outcome
1	6	M	-			dosage in plasma ^a^
2	6	M	-			dosage in brain ^a^
3M	6	M	per os ^b^			dosage in plasma
3F	6	F	per os ^b^	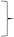		dosage in plasma
4M	6	M	per os ^b^			dosage in brain
4F	6	F	per os ^b^	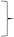		at 3 h
5M	6	M	per os ^b^			dosage in brain
5F	6	F	per os ^b^	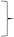		at 5 h
6M	6	M	per os ^b^			dosage in brain
6F	6	F	per os ^b^			at 7 h
7	6	M	per os ^b^	dosage in brain	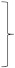	at 12 h
8	6	M	os ^b^			at 24 h
9	6	M	per os ^b^			at 48 h
10M	6	M	Iv ^c^			dosage in plasma
10F	6	F	Iv ^c^			dosage in plasma

^a^ With spiked amounts of TDMQ20 (controls). ^b^ Intragastric delivery of 25 mg/kg of TDMQ20 in 0.9 wt% aqueous NaCl (3 mg/mL, injected volume = ca. 2 mL), ^c^ Intravenous delivery of 2.5 mg/kg of TDMQ20 in 0.9 wt% aqueous NaCl (3 mg/mL, injected volume = ca. 0.7 mL).

**Table 2:** Pharmacokinetic parameters of TDMQ20 in rat plasma and brain after oral (25 mg/kg) or intravenous administration (2.5 mg/kg). Calculated by DAS 2.0 software.

Administration Route				Mean ± SD	
iv	*in plasma*	Parameter	Unit	Males ^a^	Females ^a^
		AUC_(0−t)_	µg/L·h	211 ± 33	226 ± 32
		AUC_(0−∞)_	µg/L·h	281 ± 55	355 ± 159
		MRT_(0−t)_	h	1.9 ± 0.2	1.7 ± 0.2
		t_1/2_	h	2.8 ± 1.5	3.3 ± 2.7
		CL	L/h/kg	9.2 ± 1.8	8.1 ± 2.8
Oral	*in plasma*	AUC_(0−t)_	µg/L·h	1778 ± 193	2906 ± 675
		AUC_(0−∞)_	µg/L·h	1843 ± 215	3046 ± 732
		MRT_(0−t)_	h	2.5 ± 0.4	2.4 ± 0.3
		t_1/2_	h	2.7 ± 0.3	3.3 ± 2.0
		T_max_	h	0.6 ± 0.3	0.8 ± 0.3
		CL/F	L/h/kg	13.7 ± 1.7	8.7 ± 2.5
		C_max_	µg/L	830 ± 111	1251 ± 324
		F ^b^		66%	86%
Oral	*in brain*	V_d_	L/kg	36 ± 13	30 ± 13
		AUC_(0−t)_	µg/g·h	35.5 ± 1.6 ^c^	
		AUC_(0−∞)_	µg/g·h	83.2 ± 31.8 ^c^	
		MRT_(0−t)_	h	22.0 ± 1.2 ^c^	
		t_1/2_	h	40.9 ± 16.3 ^c^	
		T_max_	h	12.8 ± 9.0 ^c^	
		CL/F	L/h/kg	0.3 ± 0.1 ^c^	
		C_max_	µg/g	1.1 ± 0.7 ^c^	

^a^ n = 6 except otherwise stated, ^b^ F (%) = [dose iv × oral AUC((0−∞)]/[dose oral × iv AUC(0−∞)] × 100, ^c^ n = 12, male/female ratio = 1/1.

## Data Availability

Not applicable.

## Supplementary Materials

The following supporting information can be downloaded at: https://www.mdpi.com/article/10.3390/pharmaceutics14122691/s1, Figure S1: (a) MS/MS Spectrum of TDMQ20 (*m*/*z* = 327.3, MH^+^). (b) Structures of TDMQ20 fragments (*m*/*z* = 239.2 and 204.2); Figure S2: Calibration curves of TDMQ20 concentration in rat plasma (a) and in rat brain (b), constructed by comparing the peak area ratio of TDMQ20/IS (Y) against TDMQ20 concentration (X) using the least squares linear regression method with 1/X weighting. Y is calculated as the area of the transition 327.3 → 239.2 of TDMQ20 divided by the area of transition 189.1 → 143.2 of IS; Figure S3: MRM chromatograms of transitions *m*/*z* 327.1 → 239.1 representative of TDMQ20 (a) or transition *m*/*z* 189.1 → 143.1 representative of IS (b) in (A) blank plasma, (B) blank plasma spiked with TDMQ20 (10 ng/mL) and IS (2000 ng/mL), (C) rat plasma sample 1.0 h after oral administration of TDMQ20 (25 mg/kg). The retention times of TDMQ20 and IS are 3.71 min and 3.86 min, respectively; Figure S4: MRM chromatograms of transitions *m*/*z* 327.1 → 239.1 representative of TDMQ20 (a) or transition *m*/*z* 189.1 → 143.1 representative of IS (b) in (A) blank rat brain homogenate, (B) blank rat brain homogenate spiked with TDMQ20 (10 ng/mL) and IS (8000 ng/mL), (C) rat brain sample 2.0 h after oral administration of TDMQ20 (25 mg/kg). The retention times of TDMQ20 and IS were 3.71 min and 3.86 min, respectively; Table S1: HPLC analyses: Mobile phase composition and gradient elution table. Eluents: (A) 0.1% formic acid in water, (B) methanol; Flow rate: 0.3 mL/min; Column temperature: 40 °C; Injection volume: 1 μL; Table S2: Mass spectrometry parameters; Table S3: Fragmentation used in MRM mode; Table S4: Intra-day and inter-day accuracy and precision of TDMQ20 (n = 5); Table S5: Extraction recovery and matrix effect of TDMQ20 in rat plasma and brain homogenate; Table S6: Stability data of TDMQ20 in rat plasma and brain homogenate; Table S7: TDMQ20 concentration in plasma (ng/mL) after intravenous administration at 2.5 mg/kg; Table S8: TDMQ20 concentration in plasma (ng/mL) after oral administration at 25 mg/kg; Table S9: TDMQ20 concentration in brain extracts (ng/g) after oral administration at 25 mg/kg.
