# Supplementary material for: Distribution in Rat Blood and Brain of TDMQ20, a Copper Chelator Designed as a Drug-Candidate for Alzheimer’s Disease
Source: Pharmaceutics. 2022 Dec 1;14(12):2691. doi: 10.3390/pharmaceutics14122691 (PMC9785178; doi:10.3390/pharmaceutics14122691)
Supplement: Supplementary file 1 [file pharmaceutics-14-02691-s001.zip › pharmaceutics-2024591-supplementary.pdf]

Distribution in rat blood and brain of TDMQ20, a copper chelator designed as a drug-candidate for Alzheimer's disease.

Lan Huang,[1] Yaoxun Zeng,[2] Yongliang Li,[1] Yingshan Zhu, [1] Yan He,[2] Yan Liu,[1*] Anne Robert,[3] Bernard Meunier[1,3*]

1) School of Chemical Engineering and Light Industry, Guangdong University of Technology, Guangzhou 510006, China.

2) School of Biomedical and Pharmaceutical Sciences, Guangdong University of Technology, Guangzhou 510006, China.

3) Laboratoire de Chimie de Coordination du CNRS (LCC-CNRS), Inserm ERL 1289, 205 route de Narbonne, 31077 Toulouse, France.

Supplementary Material

LC-MS/MS method

Table S1. HPLC analyses: Mobile phase composition and gradient elution table. Eluents: (A) 0.1% formic acid in water, (B) methanol; Flow rate: 0.3 mL/min; Column temperature: 40 °C; Injection volume:1 µL.

Table S2. Mass spectrometry parameters.

Table S3. Fragmentation used in MRM mode.

Description and validation of TDMQ20 quantification method

Figure S1. **(a)** MS/MS Spectrum of TDMQ20 (*m/z* = 327.3, MH^+). **(b)** Structures of TDMQ20 fragments (*m/z* = 239.2 and 204.2).

Figure S2. Calibration curves of TDMQ20 concentration in rat plasma **(a)** and in rat brain **(b)**, constructed by comparing the peak area ratio of TDMQ20/IS (Y) against TDMQ20 concentration (X) using least squares linear regression method with 1/X weighting. Y is calculated as area of the transition 327.3 → 239.2 of TDMQ20 divided by the area of transition 189.1 → 143.2 of IS.

Figure S3. MRM Chromatograms of transitions *m/z* 327.1 → 239.1 representative of TDMQ20 (**a**) or transition *m/z* 189.1 → 143.1 representative of IS (**b**) in (A) blank plasma, (B) blank plasma spiked with TDMQ20 (10 ng/mL) and IS (2000 ng/mL), (C) rat plasma sample 1.0 h after oral administration of TDMQ20 (25 mg/kg) The retention times of TDMQ20 and IS are 3.71 min and 3.86 min, respectively.

Figure S4. MRM Chromatograms of transitions *m/z* 327.1 → 239.1 representative of TDMQ20 (**a**) or transition *m/z* 189.1 → 143.1 representative of IS (**b**) in (A) blank rat brain homogenate, (B) blank rat brain homogenate spiked with TDMQ20 (10 ng/mL) and IS (8000 ng/mL), (C) rat brain sample 2.0 h after oral administration of TDMQ20 (25 mg/kg). The retention times of TDMQ20 and IS were 3.71 min and 3.86 min, respectively.

Table S4. Intra-day and inter-day accuracy and precision of TDMQ20 (n = 5).

Table S5. Extraction recovery and matrix effect of TDMQ20 in rat plasma and brain homogenate.

Table S6. Stability data of TDMQ20 in rat plasma and brain homogenate.

Dosage of TDMQ20 in rat plasma and brain after intravenous or oral administration

Table S7. TDMQ20 concentration in plasma (ng/mL) after intravenous administration at 2.5 mg/kg.

Table S8. TDMQ20 concentration in plasma (ng/mL) after oral administration at 25 mg/kg.

Table S9. TDMQ20 concentration in brain extracts (ng/g) after oral administration at 25 mg/kg.

LC-MS/MS method

Table S1. HPLC analyses: Mobile phase composition and gradient elution table. Eluents: (A) 0.1% formic acid in water, (B) methanol; Flow rate: 0.3 mL/min; Column temperature: 40 ℃; Injection volume:1 µL.

Time (min)	%A	%B
0	90	10
1	90	10
3	10	90
6	10	90
6.1	90	10
8	90	10

Table S2. Mass spectrometry parameters.

Mass spectrum parameters	
Ionization mode	H-ESI$^+$
Spray Voltage/V	3500
Vaporizer Temperature/℃	350
Capillary Temperature/℃	320
Sheath Gas/Arb	35
AUX Gas/Arb	10
Sweep Gas/Arb	0

Table S3. Fragmentation used in MRM mode.

Compound	Polarity	Precursor (*m/z*)	Fragment (*m/z*)	Collision Energy (V)	RF Lens (V)
TDMQ20	+	327.11	239.06	16.32	93
	+	327.11	204.04	39.58	93
IS	+	189.07	143.17	21.48	100
	+	189.07	128.17	36.44	100

Description and validation of TDMQ20 quantification method

Figure S1. (a) MS/MS Spectrum of TDMQ20 (*m/z* = 327.3, MH^+). **(b)** Structures of TDMQ20 fragments (*m/z* = 239.2 and 204.2).

(a)

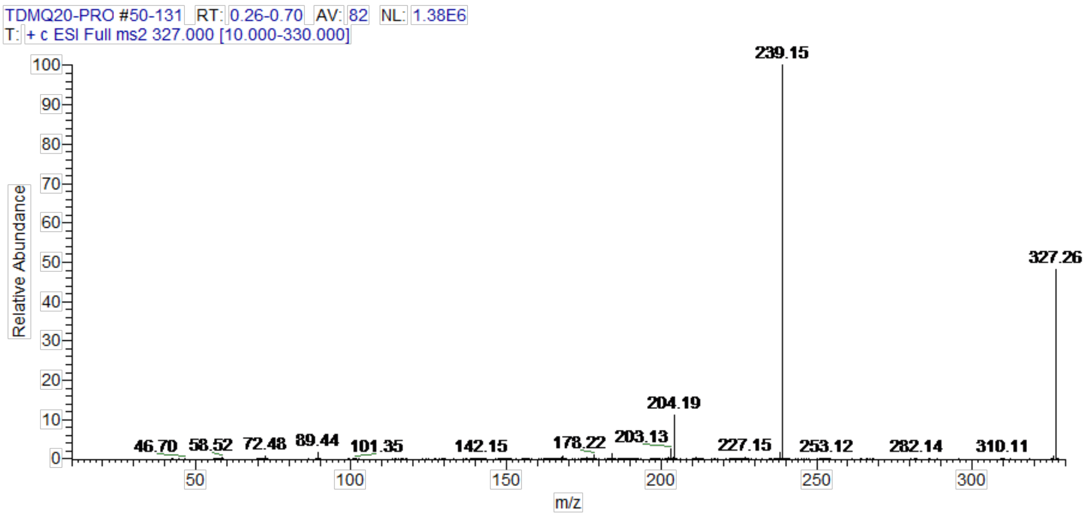

(b)

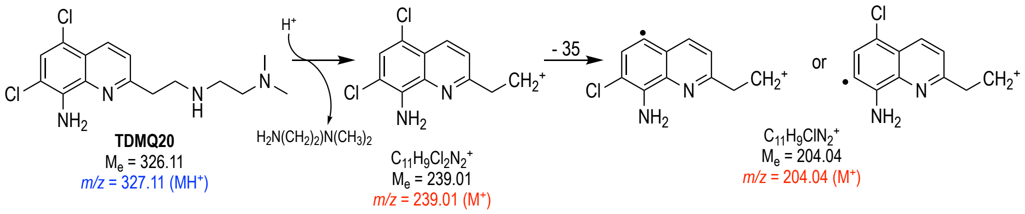

Figure S2. Calibration curves of TDMQ20 concentration in rat plasma **(a)** and in rat brain **(b)**, constructed by comparing the peak area ratio of TDMQ20/IS (Y) against TDMQ20 concentration (X) using least squares linear regression method with 1/X weighting. Y is calculated as area of the transition 327.3 → 239.2 of TDMQ20 divided by the area of transition 189.1 → 143.2 of IS.

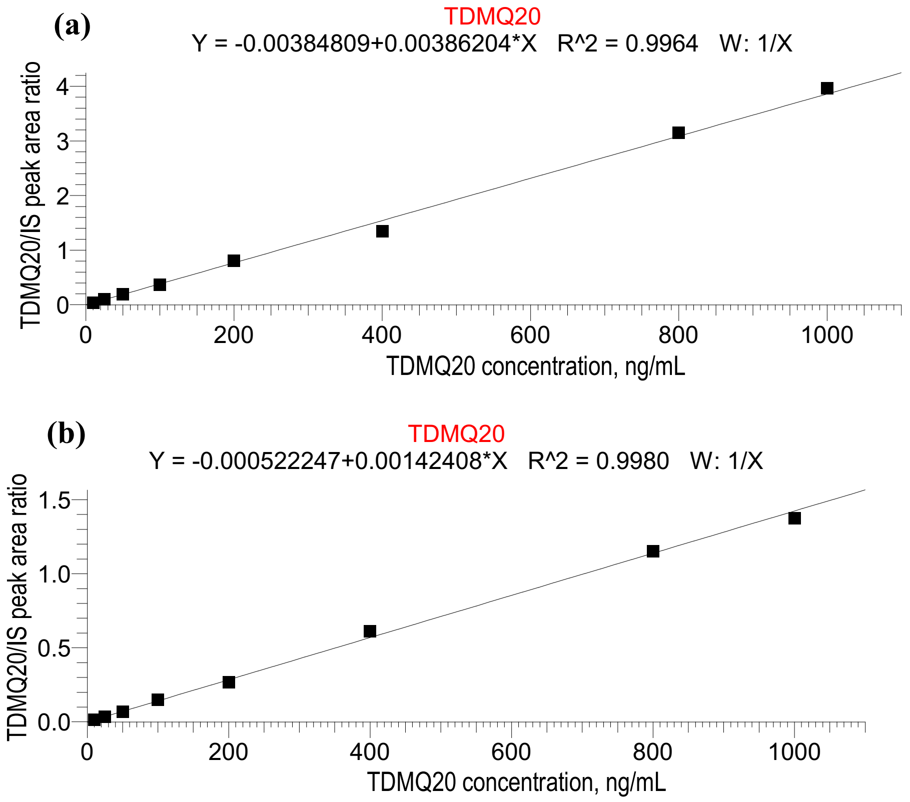

Figure S3. MRM Chromatograms of transitions *m/z* 327.1 → 239.1 representative of TDMQ20 (**a**) or transition *m/z* 189.1 → 143.1 representative of IS (**b**) in (A) blank plasma, (B) blank plasma spiked with TDMQ20 (10 ng/mL) and IS (2000 ng/mL), (C) rat plasma sample 1.0 h after oral administration of TDMQ20 (25 mg/kg) The retention times of TDMQ20 and IS are 3.71 min and 3.86 min, respectively.

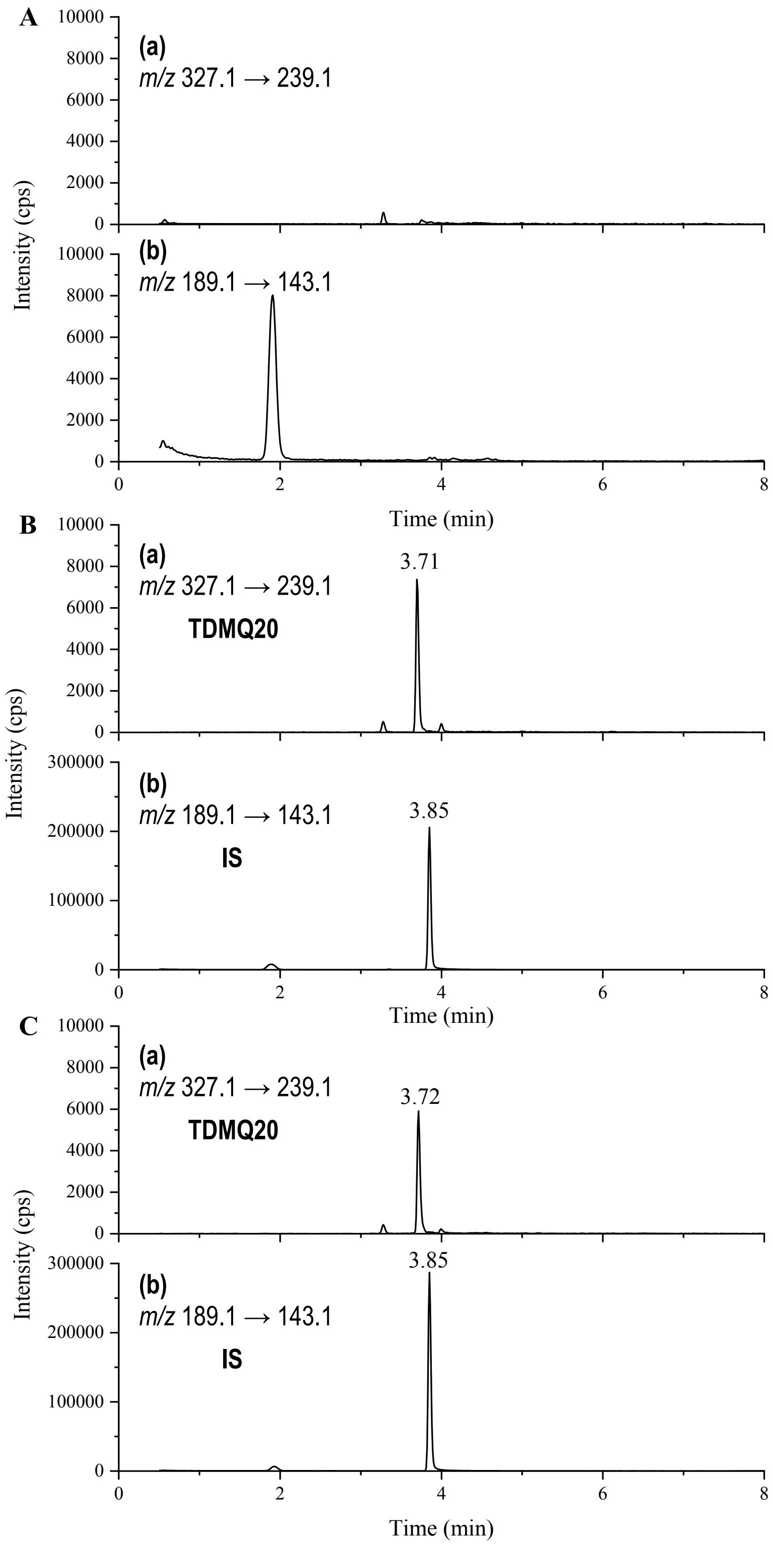

Figure S4. MRM Chromatograms of transitions *m/z* 327.1 → 239.1 representative of TDMQ20 (**a**) or transition *m/z* 189.1 → 143.1 representative of IS (**b**) in (A) blank rat brain homogenate, (B) blank rat brain homogenate spiked with TDMQ20 (10 ng/mL) and IS (8000 ng/mL), (C) rat brain sample 2.0 h after oral administration of TDMQ20 (25 mg/kg). The retention times of TDMQ20 and IS were 3.71 min and 3.86 min, respectively.

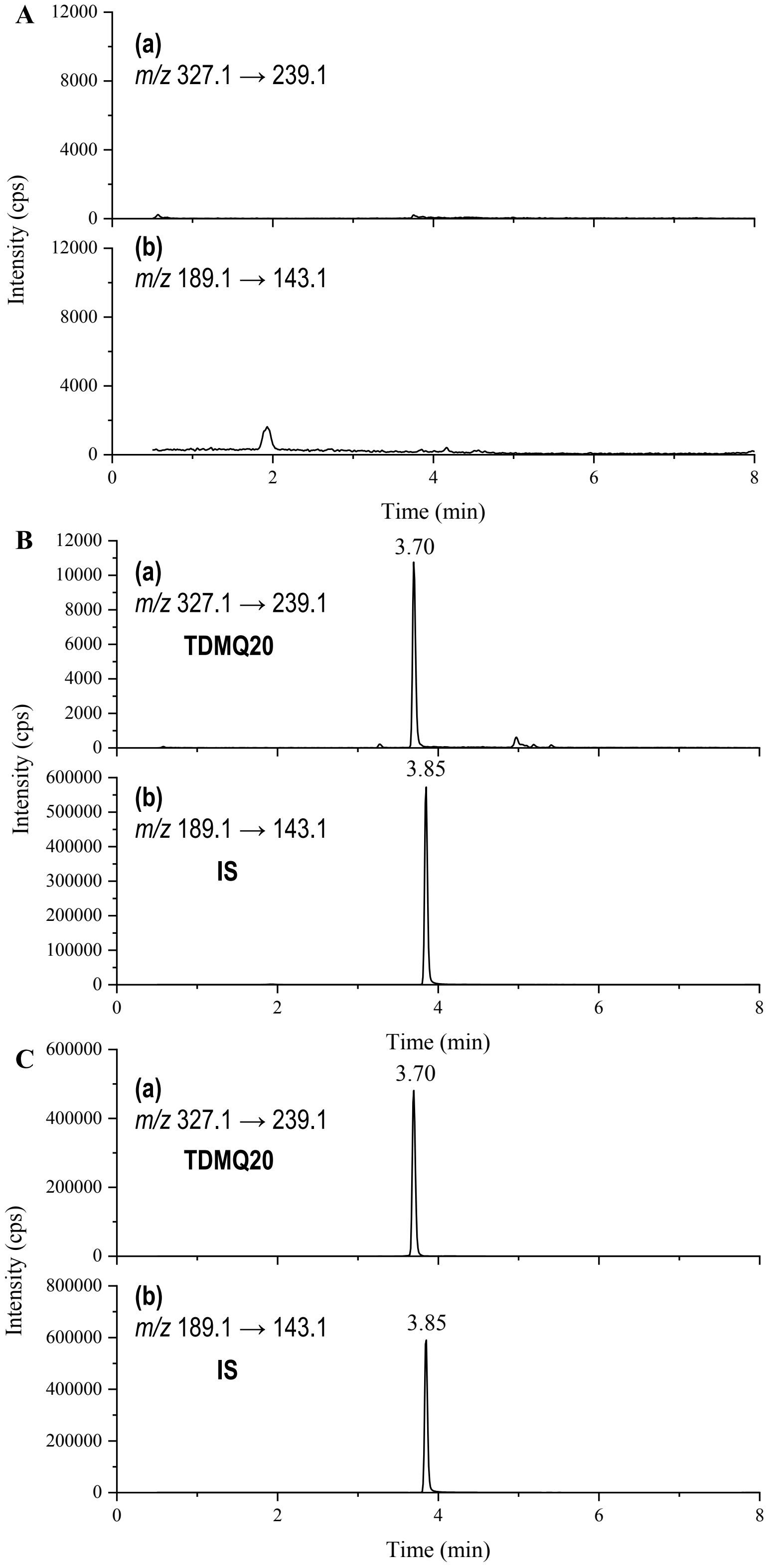

Table S4. Intra-day and inter-day accuracy and precision of TDMQ20 (n = 5).

Matrix	Spiked conc. (ng/mL)	Intra-day			Inter-day		
		Measured conc. (ng/mL)	Accuracy (RE%)[a]	Precision (CV%)	Measured conc. (ng/mL)	Accuracy (RE%)[a]	Precision (CV%)
Plasma	10	9.7	-2.7	13.2	11.0	10.2	14.5
	25	23.9	-4.5	3.1	24.2	-3.4	4.7
	500	496.6	-0.7	3.3	507.3	1.5	5.3
	750	751.9	0.3	4.4	767.9	2.4	5.8
Brain	10	10.3	3.2	8.8	10.8	8.0	9.1
	25	24.3	-2.8	3.1	24.8	-0.8	3.2
	500	480.1	-4.0	3.3	499.4	-0.1	4.2
	750	708.1	-5.6	4.4	721.1	-4.4	4.3

[a]RE% = (Measured concentration–Spiked concentration)/Spiked concentration x 100

Table S5. Extraction recovery and matrix effect of TDMQ20 in rat plasma and brain homogenate.

Matrix	Spiked conc. (ng/mL)	Extraction recovery (%)	RSD (%)	Matrix effect (%)	RSD (%)
	25	95.7	3.6	100.6	5.0
Plasma	500	93.7	7.6	96.9	5.7
	750	97.6	3.3	97.7	2.8
Brain	25	91.1	3.3	108.8	3.9
	500	93.7	2.7	98.6	2.9
	750	94.5	1.9	102.9	0.9

Table S6. Stability data of TDMQ20 in rat plasma and brain homogenate.

Matrix	Spiked conc. (ng/mL)	Room temperature for 4h		Storage at 4°C for 24h		Three freeze-thaw cycles		Storage at -20°C for 14 days	
		RE(%)[a]	RSD(%)	RE(%)[a]	RSD(%)	RE(%)[a]	RSD(%)	RE(%)[a]	RSD(%)
Plasma	25	-1.7	2.4	1.1	3.6	-1.6	5.1	-6.5	4.4
	750	1.4	5.0	4.7	1.5	6.5	3.3	6.5	3.3
Brain	25	-3.9	3.1	1.9	2.1	5.7	3.2	3.0	3.6
	750	-6.8	5.6	-7.0	1.2	-6.5	0.8	-3.7	0.3

[a]RE% = (Measured concentration- Spiked concentration)/Spiked concentration x 100

Dosage of TDMQ20 in rat plasma and brain after intravenous or oral administration

Table S7. TDMQ20 concentration in plasma (ng/mL) after intravenous administration at 2.5 mg/kg.

Individual data

Time (min)		Female						
	1f	2f	3f	4f	5f	6f	Mean	SEM
2	194	153	148	169	167	216	175	11
5	104	98	159	144	112	166	130	12
10	109	100	143	99	69	92	102	10
30	70	87	84	91	46	59	73	7
60	47	55	56	72	61	81	62	5
90	64	35	31	37	28	56	42	6
120	53	33	75	43	28	13	41	9
180	26	28	35	34	26	25	29	2
300	32	16	31	43	38	34	32	4
Time (min)		Male						
	1m	2m	3m	4m	5m	6m	Mean	SEM
2	116	190	137	-	216	69	146	24
5	89	151	105	146	162	71	121	15
10	95	121	92	96	150	70	104	11
30	62	97	96	62	88	67	79	7
60	64	74	47	40	47	47	53	5
90	57	74	44	48	55	42	53	5
120	56	29	23	24	35	34	33	5
180	52	22	24	22	27	37	31	5
300	25	16	11	17	19	15	17	2

Table S8. TDMQ20 concentration in plasma (ng/mL) after oral administration at 25 mg/kg.

Individual data

Time (min)		Females						
	1f	2f	3f	4f	5f	6f	Mean	SEM
10	546	591	376	632	316	184	441	72
20	766	1064	572	998	648	318	728	113
30	1322	1576	839	1276	676	590	1047	163
60	1580	1567	1287	1198	1062	727	1237	132
120	772	673	634	606	500	398	597	54
180	338	243	272	305	185	153	249	29
300	106	61	129	80	46	67	82	13
480	66	32	103	57	54	62	62	9
720	33	23	30	40	20	19	27	3
Time (min)		Males						
	1m	2m	3m	4m	5m	6m	Mean	SEM
10	295	355	192	262	334	562	333	51
20	520	604	491	511	608	835	595	52
30	650	655	806	721	812	785	738	74
60	870	619	804	999	642	613	758	65
120	276	245	291	136	180	213	223	24
180	150	196	142	62	49	145	124	23
300	71	125	66	51	73	126	85	13
480	47	68	47	28	34	42	44	6
720	17	26	17	10	12	14	16	2

Table S9. TDMQ20 concentration in brain extracts (ng/g) after oral administration at 25 mg/kg.

Individual data

Time (h)		Females						
							Mean	SEM
3	564	199	520	1354	1007	479	687	170
5	1099	828	717	826	441	868	797	88
7	505	1768	1996	1754	1025	1039	1348	236
Time (h)		Males						
							Mean	SEM
3	291	543	363	143	333	248	320	54
5	1138	863	511	803	1017	671	834	93
7	554	1009	731	763	807	1040	817	74
12	1111	1171	856	938	1010	771	976	62
24	810	912	1006	1001	883	908	920	30
48	514	321	467	514	478	341	439	35
Time (h)		Males + Females 1/1 (n = 12)						
							Mean	
3							504	
5							816	
7							1083	

Note. Three hours after TDMQ20 oral administration, [TDMQ20] in plasma was 249 ng/mL and 124 ng/mL for females and males, respectively (Figure 3, Table S8). At 3 h, [TDMQ20] in brain was 687 ng/g and 320 ng/g (Figure 4, Table S9). The ng/g unit can be considered roughly

equivalent to ng/mL. Consequently, the brain-plasma concentration ratios were similar for females and males, with values = 687/249 = 2.8 and 320/124 = 2.6 at 3 h, for females and males, respectively. Calculated at Cmax (ca. 12.8 h for brain and 0.6 h for plasma), the brain/plasma concentration ratio for male rats was [TDMQ20]brain, 12.8 h / [TDMQ20]plasma, 0.6 h = 1100/830 = 1.3.
